# Thrombolysis for Cardiogenic Shock Secondary to Aortic Bioprosthetic Valve-in-Valve Thrombosis

**DOI:** 10.7759/cureus.33141

**Published:** 2022-12-30

**Authors:** Jian Chu, Nikitaa Nath, Steve Attanasio

**Affiliations:** 1 Internal Medicine, Rush University Medical Center, Chicago, USA; 2 Cardiovascular Medicine, Rush University Medical Center, Chicago, USA

**Keywords:** valve-in-valve, tissue plasminogen activator (tpa), cardiogenic shock, systemic thrombolysis, bioprosthetic aortic valve thrombosis

## Abstract

Valvular obstruction and thromboembolism are feared complications of bioprosthetic valve thrombosis. We describe the case of an 81-year-old man with a prior aortic valve-in-valve bioprosthesis who presented in cardiogenic shock, requiring mechanical circulatory support. He was found to have acute bioprosthetic valve thrombosis and was treated with systemic thrombolysis. This case highlights the overall uncertainty regarding the optimal treatment of acute bioprosthetic valve thrombosis. Society guidelines and the current evidence behind prophylaxis and treatment are reviewed. Although the data remain sparse, systemic thrombolysis may be an effective strategy in critically ill patients who are poor surgical candidates.

## Introduction

Aortic stenosis (AS) is the most common cardiac valvulopathy in developed countries with an estimated prevalence of about 4% in individuals 70-79 years of age [1]. With increasing global life expectancy and improved transcatheter outcomes, the number of elderly patients undergoing aortic valve replacement (AVR) has increased substantially in the past decade [2,3]. Bioprosthetic valve thrombosis (BPVT) is a feared complication of AVR that can lead to fatal hemodynamic compromise and thromboembolic events. In this report, we describe a case of cardiogenic shock due to aortic BPVT that was treated with systemic thrombolysis.

## Case presentation

An 81-year-old man with an aortic valve-in-valve bioprosthesis presented to the emergency department from home with weakness and acute dyspnea. On exam, he was afebrile with a blood pressure of 80/50 mm Hg, heart rate of 105 beats/min, respiratory rate of 23 breaths/min, and saturating 95% on room air. Physical exam was notable for mild respiratory distress, left basilar crackles, and cold lower extremities.

He had an extensive cardiac history that was significant for coronary artery disease with a single-vessel coronary artery bypass graft and percutaneous coronary intervention, heart failure with reduced ejection fraction (left ventricular ejection fraction (LVEF) 25%) with cardiac resynchronization therapy (CRT-D), atrial flutter ablation, mitral annuloplasty ring, and surgical aortic valve replacement with a 27 mm Edwards pericardial valve (Edwards Lifesciences, Irvine, California) followed by subsequent valve-in-valve transcatheter aortic valve replacement (VIV TAVR) with a 26-mm Edwards SAPIEN 3 prosthetic valve (Edwards Lifesciences) that was performed five years prior to presentation.

During routine follow-up, he was found to have increased aortic transvalvular gradients to 47 mmHg on echocardiography. Cardiac computed tomography revealed restricted valve opening and leaflet thickening, consistent with valve thrombosis with possible pannus formation. He was subsequently started on warfarin with a significant improvement in the mean gradient from 47 to 16 mmHg, but warfarin was discontinued 18 months prior to presentation due to ocular bleeding. At the time of discontinuation, a shared decision was made to monitor closely with clinical assessments and surveillance echocardiograms. He was seen in the cardiology clinic two months ago for a routine follow-up with New York Heart Association (NYHA) Class I functional status.

His basic metabolic panel was remarkable for a blood urea nitrogen of 74 mg/dL and serum creatinine of 1.86 mg/dL (baseline Cr 1.3-1.4 mg/dL). Liver enzymes and complete blood counts were normal, except for a white blood cell count of 15,500/uL. Venous lactate was 2.1 mmol/L. High-sensitivity troponin was >50,000 ng/L, which exceeded the upper limit of detection and B-type natriuretic peptide was 3,056 pg/mL. The chest X-ray was remarkable for mild to moderate volume overload changes. The electrocardiogram (ECG) demonstrated biventricular pacing with ST elevations in leads II, III, and aVF that did not meet Sgarbossa criteria. Bedside transthoracic echocardiography (TTE) showed markedly reduced left ventricular function with an estimated LVEF of 10-15%.

Due to high suspicion of cardiogenic shock, the patient underwent emergent right and left heart catheterization. No flow-limiting coronary artery or bypass graft disease was evident. Hemodynamics were notable for an elevated pulmonary capillary wedge pressure of 21 mmHg and mean pulmonary artery pressure of 34 mmHg. Cardiac output by thermodilution was 3.3 L/min and the cardiac index was 1.6 L/min/m^2^. He was started on dobutamine 5 mcg/kg/min as well as norepinephrine and vasopressin. An intra-aortic balloon pump (IABP) was inserted and a Swan-Ganz catheter was left in place prior to admission to the cardiac ICU.

**Figure 1:**
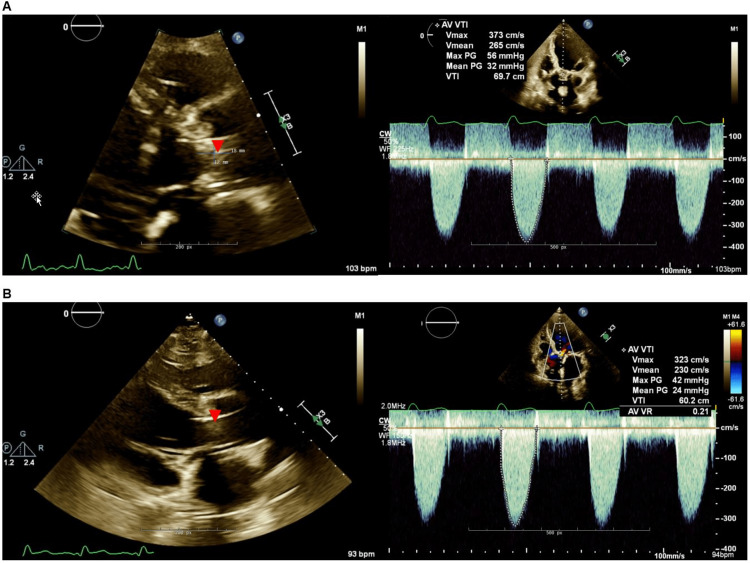
(A) Echocardiogram demonstrating a bioprosthetic aortic valve thrombus (red arrowhead) measuring 12 mm x 18 mm in parasternal long axis view with Doppler mode demonstrating a peak systolic velocity of 3.7 meters/second (m/s) and a mean gradient of 32 mmHg. (B) Echocardiogram after treatment showing improvement in the size of valvular thrombus (red arrowhead) associated with a decrease in peak systolic velocity to 3.2 m/s and mean gradient to 24 mmHg

A transthoracic echocardiogram demonstrated an LVEF of 10-15%, severe diffuse LV hypokinesis, intact mitral valve annuloplasty ring without stenosis, and mild mitral regurgitation. This study was notable for the dysfunction of the prosthetic aortic valve with a peak velocity of 3.7 m/s and a mean gradient of 32 mmHg. Upon further review of the images, echodensity on the bioprosthetic aortic valve leaflet measuring 12 x 18 mm was evident (Figure 1A) raising concern for aortic bioprosthetic valve thrombosis (BPVT) that was contributing to cardiogenic shock. Although his lactic acidosis and acute kidney injury improved with inotropic and mechanical support, the patient was ultimately unable to wean support. Cardiothoracic surgery and interventional cardiology evaluated the patient and determined he was at high risk for surgical thrombectomy. After a multidisciplinary discussion, the patient and his family elected systemic thrombolysis with a tissue plasminogen activator (tPA). He was given a load of tPA 10 mg followed by 40 mg over four hours.

The patient tolerated thrombolysis with minor bleeding diathesis, including hematuria and epistaxis. Over the 24 hours after tPA administration, the patient was able to wean off all pressors, and dobutamine was reduced from 5 to 2.5 mcg/kg/min. A repeat echocardiogram re-demonstrated prosthetic valve thrombus with improved flow parameters with decreased aortic valve peak velocity (3.7 to 3.2 m/s) and mean gradient (32 to 24 mm Hg) (Figure 1B). Unfortunately, the patient suffered recurrent episodes of pulseless ventricular tachycardia (VT) 48 hours after thrombolysis, likely related to myocardial ischemia. Due to a malfunction of his implantable defibrillator device, advanced cardiac life support (ACLS) was initiated without return of spontaneous circulation. The family ultimately decided to stop resuscitative efforts, and the patient passed away.

## Discussion

The estimated incidence of BPVT ranges from 0.5-7.0% with a median onset of six months postoperatively during the early stages of device endothelialization [4,5]. Patients with BPVT commonly present with acute-onset dyspnea secondary to valvular dysfunction or thromboembolic events. The diagnosis of BPVT is generally based on the imaging findings of valve leaflet thickening and reduced leaflet mobility associated with increased transvalvular gradient (≥ 20 mmHg), reduced orifice area, or new-onset valvular regurgitation. American and European Society guidelines recommend anticoagulation for risk reduction of early thromboembolism. Although there are slight differences in recommended agents, vitamin K antagonists (VKAs) are generally favored post-SAVR, and dual antiplatelet or aspirin monotherapy is favored post-TAVR [6,7]. Some observational studies demonstrate that the increased risk of thromboembolism extends beyond this early period [8], prompting consideration for lifelong aspirin monotherapy.

New data suggest that long-term BPVT is more common than previously thought. The recent PARTNER 3 trial and SAVORY registry highlight subclinical leaflet thrombosis as a relatively common late finding in up to one-quarter of patients at one-year follow-up after AVR [9,10]. However, it is uncertain what proportion of these patients go on to develop clinically significant BPVT. One single-center retrospective study estimated an event rate of clinically significant BPVT at 1.5 per 100 patient-years of follow-up with 17% of cases occurring after one year postoperatively [11]. This study, along with the Valve-in-Valve International Data Registry [12], highlights the higher risk for BPVT with valve-in-valve (VIV) procedures. These findings suggest a benefit to preventive anticoagulation for patients with risk-enhancing factors that include VIV procedures, hypercoagulopathy, paroxysmal atrial fibrillation, and recurrent thromboses.

The optimal management of acute BPVT is unknown and lacks the support of randomized controlled trials. Early referral to a multidisciplinary valve team composed of a cardiovascular imaging specialist, interventional cardiologist, and cardiothoracic surgeon is universally recommended [7]. In hemodynamically stable patients, treatment with a VKA (e.g., coumadin or phenprocoumon) has proven a promising strategy in non-randomized prospective studies [8,11] and may be superior to direct-acting oral anticoagulants (DOACs) based on indirect findings from the GALILEO trial [13]. In this particular case, the patient had a prior diagnosis of BPVT that responded favorably to warfarin therapy with a target international normalized ratio (INR) of 2-3. However, Warfarin was subsequently discontinued at the discretion of his cardiologist due to ocular bleeding with a plan for close surveillance. Other therapeutic considerations include the off-label use of DOACs or adjustment of the INR goal. Although DOACs have not been directly compared against warfarin for BPVT in the general population, there are growing data showing the non-inferiority of DOACs for the prevention of thromboembolism in post-TAVR patients with comorbid atrial fibrillation with a slight signal toward a reduced risk of major bleeding. Given the overall ease of administration, the potential application of DOACs for treating BPVT has considerable interest and underscores the need for further comparative research.

In decompensated patients with BPVT, the evidence behind the choice of treatment (surgery versus thrombolysis) is significantly weaker as reflected by current society guidelines (Table 1). The European Society of Cardiology recommends surgical valve replacement as first-line therapy for obstructive mechanical valve thrombosis [7], whereas the American College of Chest Physicians recommends surgery based on the thrombus area [14]. The American College of Cardiology and the American Heart Association emphasizes the individualization of treatment for mechanical valve thrombosis based on patient factors and local expertise [6]. However, these guidelines do not comment on the use of systemic thrombolysis for BPVT in decompensated patients. Two recent systematic reviews and meta-analyses of observational studies yielded mixed results but generally found thrombolysis to be non-inferior to surgery [15,16].

**Table 1:** Current major society guidelines for the treatment of left-sided mechanical and bioprosthetic valve thrombosis in decompensated patients Abbreviations – ACC/AHA: American College of Cardiology/American Heart Association, ACCP: American College of Chest Physicians, EACTS: European Association for Cardio-Thoracic Surgery, ESC: European Society of Cardiology, UFH: unfractionated heparin, VKA: vitamin K antagonist

Statements	Mechanical	Bioprosthetic
2021 ESC/EACTS [7]	Recommends emergency valve replacement for obstructive physiology and non-obstructive thrombosis with large thrombus or embolic events	Recommends VKA or UFH as first-line treatment to be continued indefinitely after the first episode
2020 ACC/AHA [6]	Recommends individualized decision-making regarding surgery and systemic thrombolysis with consideration for patient factors and local expertise	Recommends VKA treatment as first-line treatment
2012 ACCP [14]	Recommends early surgery over systemic thrombolysis for patients with a large thrombus area (>0.8 cm^2^) unless contraindications to surgery exist. No distinction is made between the management of mechanical and bioprosthetic valves.	

The evidence for thrombolysis in aortic BPVT is largely derived from observational studies of patients with left-sided mechanical valve thrombosis. The TROIA and PROMETEE prospective observational studies showed that an ultraslow infusion of tPA - 25 mg of alteplase over 25 hours without a bolus - improved echocardiographic parameters and clinical symptoms in the majority of patients and was associated with low risk for complications and mortality compared to faster infusions [17,18]. However, the study cohorts enrolled younger and less acute patients (i.e., hemodynamically stable with predominantly NYHA Class I/II functional status). The overall efficacy and risk profile of ultraslow tPA administration are likely to differ in patients who present in extremis, a clinical situation that may justify more aggressive thrombolysis. Several successful cases of thrombolysis for aortic BPVT with faster infusions have also been reported, although complications included coronary embolism and bleeding [19,20]. The collective evidence suggests that thrombolysis may be an effective treatment option for patients with obstructive aortic BPVT with limited surgical outcomes. However, more research is needed to determine optimal thrombolytic administration.

## Conclusions

Bioprosthetic valve thrombosis is a feared complication of TAVR that can lead to cardiogenic shock. Risk factors include an existing VIV prosthesis, hypercoagulability, and arrhythmia. In select patients with contraindications to surgery, systemic thrombolysis may be an effective option. However, further research is needed to determine the optimal thrombolytic protocol.
